# A Starvation-Based 9-mRNA Signature Correlates With Prognosis in Patients With Hepatocellular Carcinoma

**DOI:** 10.3389/fonc.2021.716757

**Published:** 2021-11-26

**Authors:** Dengliang Lei, Yue Chen, Yang Zhou, Gangli Hu, Fang Luo

**Affiliations:** ^1^ Department of Hepatobiliary Surgery, The First Affiliated Hospital of Chongqing Medical University, Chongqing, China; ^2^ Central Laboratory, The First Affiliated Hospital of Chongqing Medical University, Chongqing, China

**Keywords:** starvation, gene set enrichment analysis, hepatocellular carcinoma, mRNA signature, EIF2S1

## Abstract

**Background:**

Hepatocellular carcinoma (HCC) is one of the world’s most prevalent and lethal cancers. Notably, the microenvironment of tumor starvation is closely related to cancer malignancy. Our study constructed a signature of starvation-related genes to predict the prognosis of liver cancer patients.

**Methods:**

The mRNA expression matrix and corresponding clinical information of HCC patients were obtained from the International Cancer Genome Consortium (ICGC) and The Cancer Genome Atlas (TCGA). Gene set enrichment analysis (GSEA) was used to distinguish different genes in the hunger metabolism gene in liver cancer and adjacent tissues. Gene Set Enrichment Analysis (GSEA) was used to identify biological differences between high- and low-risk samples. Univariate and multivariate analyses were used to construct prognostic models for hunger-related genes. Kaplan-Meier (KM) and receiver-operating characteristic (ROC) were used to assess the model accuracy. The model and relevant clinical information were used to construct a nomogram, protein expression was detected by western blot (WB), and transwell assay was used to evaluate the invasive and metastatic ability of cells.

**Results:**

First, we used univariate analysis to identify 35 prognostic genes, which were further demonstrated to be associated with starvation metabolism through Kyoto Encyclopedia of Genes and Genomes (KEGG) and Gene Ontology (GO). We then used multivariate analysis to build a model with nine genes. Finally, we divided the sample into low- and high-risk groups according to the median of the risk score. KM can be used to conclude that the prognosis of high- and low-risk samples is significantly different, and the prognosis of high-risk samples is worse. The prognostic accuracy of the 9-mRNA signature was also tested in the validation data set. GSEA was used to identify typical pathways and biological processes related to 9-mRNA, cell cycle, hypoxia, p53 pathway, and PI3K/AKT/mTOR pathway, as well as biological processes related to the model. As evidenced by WB, EIF2S1 expression was increased after starvation. Overall, EIF2S1 plays an important role in the invasion and metastasis of liver cancer.

**Conclusions:**

The 9-mRNA model can serve as an accurate signature to predict the prognosis of liver cancer patients. However, its mechanism of action warrants further investigation.

## Introduction

Liver cancer is a type of cancer with the highest incidence worldwide, and hepatocellular carcinoma (HCC) accounts for 80% of liver cancer cases (1). Despite advances in treatments such as surgery, ablation, and liver transplantation (2), liver cancer remains one of the leading causes of death among all cancers (3, 4). Furthermore, increases in non-alcoholic fatty liver disease (NAFLD), metabolic syndrome, and obesity elevated the risk of liver disease (5).

Therefore, the identification of new biological molecular predictors to improve the prognosis of HCC is urgent.

The tumor microenvironment is mainly composed of hematopoietic and mesenchymal cells, as well as non-cellular components (6). it is closely associated with disease progression, local resistance, immune escape, and metastasis (7). Malnutrition is one of the most common conditions in tumor microenvironments due to increased nutrient depletion in cancer cells and inadequate vascular supply (8, 9). Starvation is reportedly associated with epithelial-mesenchymal transition (EMT), angiogenesis, and autophagy (10–12). For instance, in bladder cancer, starvation can induce autophagy in cancer cells, thereby enhancing the EMT of bladder cancer through the TGF pathway (13). Studies have reported that starvation can induce invasion and metastasis of HCC cells (14). However, studies on the characteristics of starvation-related malignancies in HCC survival are still lacking.

In this study, we first established a hunger-related 9-mRNA independent prognostic model using TCGA and verified the model accuracy in the ICGC database. In addition, we constructed a nomogram to assess clinical significance using risk scores and clinical factors. We then analyzed the typical pathways and biological processes associated with the 9-mRNA model through GSEA. Finally, we found that the expression and phosphorylation of the core model gene EIF2S1 were increased under starvation induction, which induced autophagy to increase EMT in HCC.

## Methods

### Data Collection

RNA expression data and related clinical information were obtained from TCGA (https://cancergenome.nih.gov/). A total of 424 samples in TCGA-LIHC were used in the following study as a training cohort (**Supplementary File 1**). In addition, data from 231 HCC patients from ICGA-LIRI-JP (https://dcc.icgc.org/) were downloaded as an independent, external validation cohort (**Supplementary File 2**). This research strictly follows TCGA and ICGC access rules and publication guidelines. Detailed information is shown in **Table 1**. The starvation-related gene set was obtained from Gene Set Enrichment Analysis (GSEA) ‘GOBP RESPONSE TO STARVATION ‘ in The Molecular Signatures Database(https://broadinstitute.org/gsea/msigdb/). It contained 196 genes responsible for the changes in the state/activity of a cell/organism as a result of a starvation stimulus (**Supplementary File 3**).

**Table 1:** Summary of baseline clinical pathological parameters of patients with HCC in the two datasets.

Characteristic	train	test
Age (years)		
≤ 65	227	98
> 65	122	162
Gender		
Male	239	192
Female	110	68
Grade		
1	45	N/A
2	171	N/A
3	120	N/A
4	13	N/A
Stage		
I	173	40
II	85	117
III	86	80
IV	5	23
Survival status		
Alive	236	214
Deceased	113	56

HCC, hepatocellular carcinoma; N/A, not applicable.

### Construction and Validation of a Signature

In the training set, we first identified 142 differentially expressed genes associated with starvation metabolism in 374 samples using R (P < 0.05) (**Supplementary File 4**). Univariate Cox regression analysis (P < 0.05) was used to obtain 39 genes associated with prognosis. Finally, multivariate Cox regression analysis was used to construct a signature containing nine genes, detailed in **Table 2**. The risk score for each patient was calculated using the following equation: risk score = (β1 × expression of gene1) + (β2 × expression of gene2) + … + (β9 × expression of gene9). All patients were divided into high- and low-risk groups based on the median risk score. Kaplan-Meier survival curve and two-sided log-rank test were used to compare the overall survival (OS) of the high- and low-risk group patients. Receiver-operating characteristic (ROC) curves were applied to assess the diagnostic efficacy of each clinicopathological characteristic and the prognostic signature. Stratified survival analysis was performed to examine the accuracy of the prognostic signature in predicting patient survival outcomes. Furthermore, we performed univariate and multivariate Cox regression analyses to evaluate whether the risk score was independent in determining the prognosis of the HCC patients. The M and N stages were not analyzed because data were missing for several patients. P < 0.05 was considered statistically significant.

**Table 2:** The information of nine mRNAs associated with overall survival in patients with HCC.

mRNA	Coef	HR	p-value	Risk
EHMT2	-0.039384857	0.961380644	P=0.005	Low
HNRNPL	-0.054911705	0.946568721	P<0.001	Low
EIF2S1	0.119336909	1.126749466	P<0.001	High
PPARGC1A	-0.058903674	0.942797581	P=0.004	Low
RRP8	0.401757994	1.494449623	P<0.001	High
FOXK1	0.270963075	1.311226652	P<0.001	High
CAD	0.160686777	1.174317088	P<0.001	High
FOXK2	0.101894034	1.107266133	P<0.001	High
MYBBP1A	-0.089665498	0.914236948	P=0.002	Low

HCC, hepatocellular carcinoma.

The mRNA expression profile matrix of 231 HCC patients from ICGC was used as an external independent validation cohort to validate the accuracy of the 9 gene signature.

### Functional Enrichment Analysis

The biological processes, molecular functions, and cell component Gene Ontology (GO) of mRNAs associated with survival were identified using GO enrichment analysis. The main signaling pathways of mRNA regulation were identified using the Kyoto Encyclopedia of Genes and Genomes (KEGG) pathway analysis.

### Establishment and Assessment of the Nomogram

We constructed a nomogram by integrating clinicopathologic characteristics, such as age, stage, sex, and grade, as well as the risk score derived from the prognostic signature to analyze the probable 3-year and 5-year OS of the patients with HCC. Calibration charts were used to evaluate the performance of the Nomogram.

### GSEA

GSEA software 4.0.1 was used to identify starvation-related gene sets in 50 HCC tissues and their adjacent tissues. Patients with HCC were divided into low- and high-risk groups based on the median risk value. GSEA was used to further analyze gene expression differences between the high- and the low-risk resistance groups. The Hallmark gene sets (h.all.v7.4.symbols.gmt), KEGG gene sets (c2.cp.kegg.v7.4.symbols.gmt) and GO gene sets (c5.go.v7.4.symbols.gmt) were downloaded from the Molecular Signatures Database (https://www.gsea-msigdb.org/gsea/msigdb/genesets.jsp). The gene sets were filtered using the maximum and minimum gene set sizes of 500 and 15 genes, respectively. The enriched gene sets were obtained based on a P-value < 0.05 and a false discovery rate (FDR) < 0.25 after performing 1,000 permutations.

### Cell Culture

Liver cancer cells (Hep-3B and Huh-7) were obtained from the Institute of Chinese Academy of Sciences. Hep-3B and Huh-7 cell lines were cultured in DMEM (Corning Incorporated, Corning, NY, USA) supplemented with 10% fetal bovine serum (FBS, Gibco, Thermo Scientific, Waltham, MA, USA). All cells were cultured in an incubator with an atmosphere of 95% O_2_ and 5% CO_2_ at 37°C.

### Western Blot

Total protein was extracted with RIPA lysis buffer (Beyotime Institute of Biotechnology, Jiangsu, China) containing protease and phosphatase inhibitors. The protein concentration was detected using a BCA protein detection kit (Jiangsu Beyotime Biotechnology Research Institute). Equivalent proteins were separated on 10% SDS-PAGE gels and then transferred to polyvinylidene fluoride (PVDF) membranes. After blocking with skimmed milk (dissolved in TBST) for 2 h, the membranes were subsequently probed using antibodies against B-actin (Cell Signaling Technology, USA, 1:10000), EIF2S1 (Cell Signaling Technology, USA, 1:1000),p-EIF2S1 (Cell Signaling Technology, USA, 1:1000), Vimentin(Cell Signaling Technology, USA, 1:1000) and E-cadherin(Cell Signaling Technology, USA, 1:1000) overnight at 4°C. The membranes were then washed with Tris-buffered saline containing Tween and incubated with an HRP‐conjugated anti-rabbit antibody at 37°C for 1 h. Finally, the protein bands on the membranes were observed with an Odyssey Scanning System.

### Small Interfering RNA Transfection

Small interfering RNA (siRNA) was purchased from GenePharma Biological Technology (Shanghai, China). Lipofectamine 2000 was transfected according to the manufacturer’s protocol. Cells were transfected with EIF2S1 siRNAs (siRNA-1: sense, GCCAUAAUCGUCCUCACCA; siRNA-2: sense, CCAUAAUCGUCCUCACCAA, siRNA-2: sense, CCAUAAUCGUCCUCACCAA) at a concentration of 50 nM for 6 h. After 48 h, the treated cells were collected for subsequent experiments.

### Immunofluorescence (IF)

The cells were immobilized with 4% paraformaldehyde, planted evenly on a slide, infiltrated with TRITON, then sealed with goat serum. The primary and fluorescent secondary antibodies were incubated overnight. Finally, nuclei were counterstained with DAPI.

### Transwell Assay

The ability of cells to migrate and invade was analyzed using a transwell chamber. A total of 8×104 cells were directly and uniformly distributed in the wells in serum-free medium for the migration experiment. Similarly, the invasion test was performed almost identically to the migration test, except that the upper chamber was first coatedwith the matrix according to the manufacturer’s instructions. For both tests, medium containing 15% FBS was added to the lower chamber as a chemical attractant. Cells were incubated in 5% CO_2_ for 8 h (migration) or 12 h (invasion). The membrane was wiped with a cotton swab; cells were removed from the upper surface of the cavity, fixed with 4% formaldehyde, and stained with 0.5% crystal violet.

### Statistical Analysis

Statistical analyses were conducted using GraphPad Prism 5.0 (San Diego, CA). The data were processed using the PERL programming language (version 5.30.2, http://www.perl.org). All statistical analyses were performed using R (version 3.6.2, https://www.r-project.org/). P < 0.05 was regarded as statistically significant.

## Results

### Identification of Starvation-Related Genes

**Figure 1:**
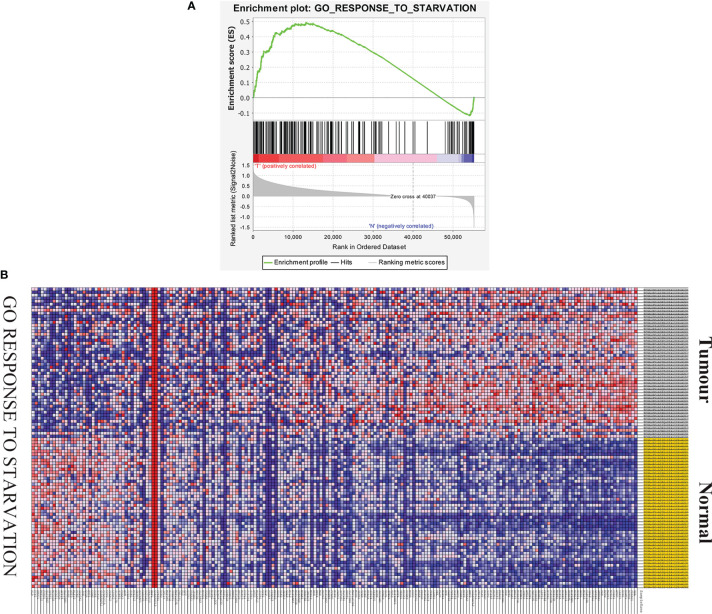
GSEA of starvation-related gene sets. **(A)** Enrichment map of one starvation-related gene set between liver cancer and paired adjacent tissues identified by GSEA. **(B)** Heat map of 196 genes in liver cancer and normal tissue response to starvation gene sets.

GSEA was used to determine whether there were significant differences in the starvation-related gene set between HCC samples and paired adjacent normal samples. The results suggested that the starvation-related gene set was significantly enriched in HCC samples (NES = 1.64, nominal P < 0.001, FDR < 0.001) (**Figure 1A**). A total of 196 starvation-related genes were used in the following study (**Figure 1B**).

### Identification of Differential Starvation-Related Genes Associated With Prognosis in Patients with HCC

**Figure 2:**
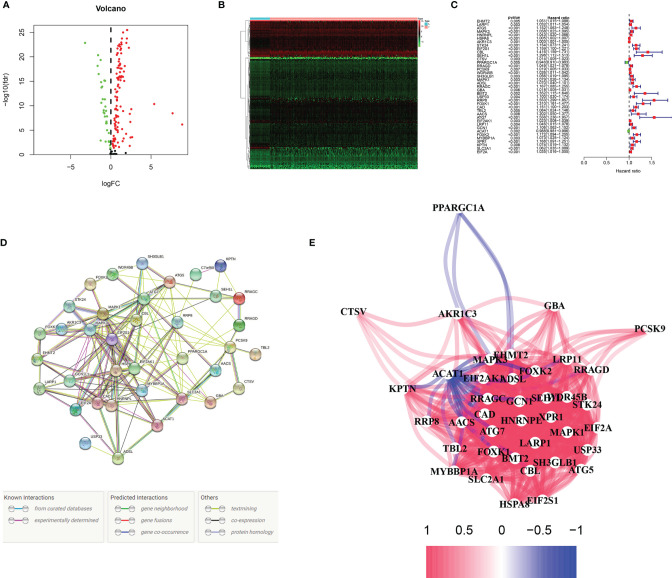
Identification of prognostic mRNAs. **(A, B)** Volcanic and heat maps of starvation-related differential genes in TCGA. P < 0.05. **(C)** Thirty-nine genes associated with patient prognosis. **(D, E)** Interaction between 39 patient prognosis-related genes.

In TCGA database, we first identified differentially expressed genes related to hunger (P < 0.05). As shown in **Figures 2A, B**, 32 of the 142 differentially expressed genes were downregulated, and 110 were upregulated. Then, we identified 39 prognostic differential genes using the univariate Cox regression analysis, among which 37 had a positive and 2 had a negative correlation with risk (**Figure 2C**). It can be seen from the hunger-related prognostic gene protein interaction network in **Figure 2D** that EIF2S1 is at the core-site. The correlation of hunger-related prognostic genes is shown in **Figure 2E**.

### KEGG and GO Analysis

**Figure 3:**
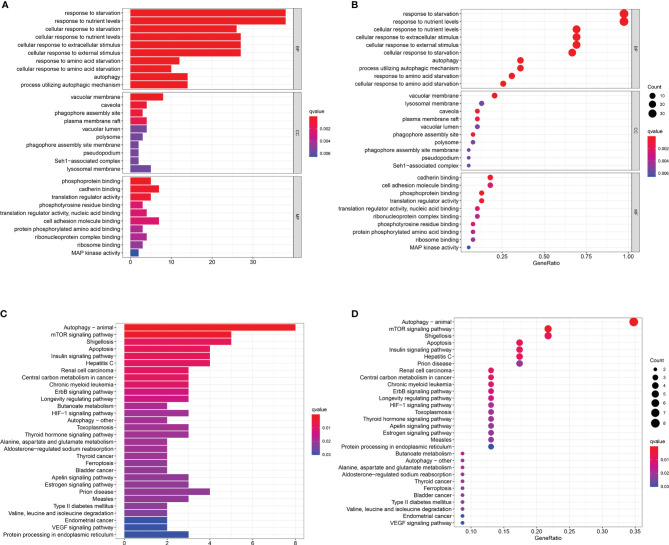
Functional enrichment analyses. **(A–D)** Gene Ontology (GO) analysis and Kyoto Encyclopedia of Genes and Genomes (KEGG) pathway analysis results showing the functions and enriched signaling pathways associated with the starvation-related mRNAs.

GO analysis and KEGG pathway enrichment analysis were used to verify whether the genes screened were involved in hunger-related energy metabolism. As shown in **Figures 3A, B**, the most notable correlation in GO is related to starvation metabolism. The same conclusion was obtained by KEGG pathway enrichment analysis (**Figures 3C, D**). GO analysis and KEGG enrichment analysis further verified that our candidate genes were closely related to hunger metabolism.

### Construction and Validation of a Starvation-Related Gene Prognostic Signature

**Figure 4:**
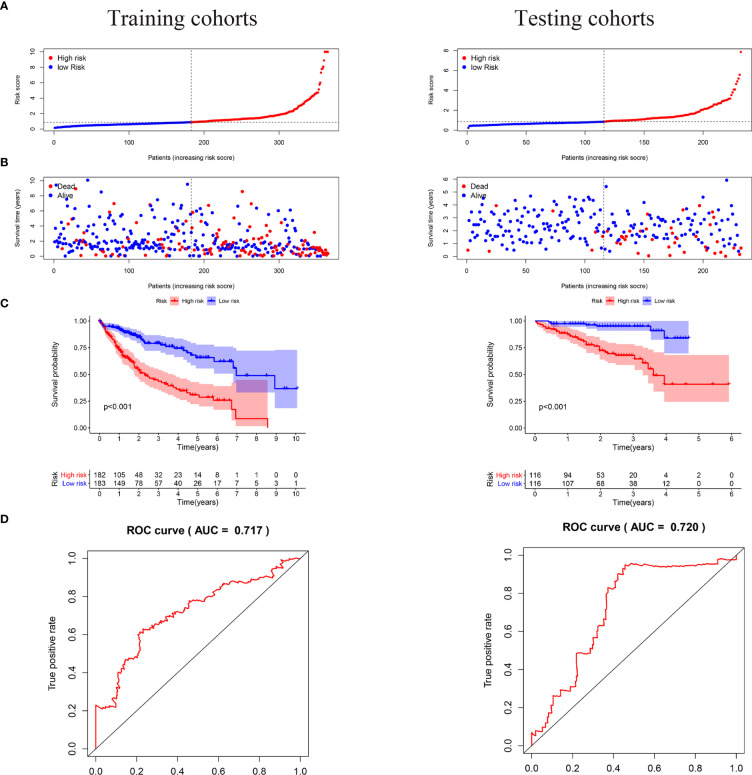
In the training and validation cohorts, the risk score based on the 9-mRNA signature predicted the OS of patients with liver cancer. **(A, B)** Risk distribution and survival status of each patient according to the 9-mRNA signature. **(C)** In the training and validation cohort, Kaplan-Meier curves showed survival outcomes for the high- and low-risk groups. **(D)** Time-dependent ROC curve of the 5 years OS was predicted with the 9-mRNA signature in the training and validation sets.

We carried out a multivariate analysis of the 39 genes obtained above and obtained 9 genes: EHMT2, HNRNPL, EIF2S1, PPARGC1A, RRP8, FOXK1, CAD FOXK2, and MYBBP1A. Among these genes were four protective genes, those with HR < 1, and five potentially harmful genes, those with HR > 1 (**Table 2**). We built a signature based on these nine genes and calculated the risk score for each patient based on the resulting model. Based on the median risk score, we divided the patients into low- and high-risk groups (**Figure 4A**). According to the graph, the number of patient deaths increases with increased risk values in the training and validation sets (**Figure 4B**). As can be seen from the training and validation sets, there were significant differences in OS between the high- and the low-risk groups (P < 0.001 and P < 0.001, respectively), indicating a higher mortality rate in the high-risk group (**Figure 4C**). ROC was used to validate the model; the AUC values for 5-year survival for the training and validation cohorts were 0.73 and 0.76, respectively, demonstrating the high accuracy of this model (**Figure 4D**).

### Starvation-Related mRNA Signature as an Independent Predictor of Survival in HCC Patients

**Figure 5:**
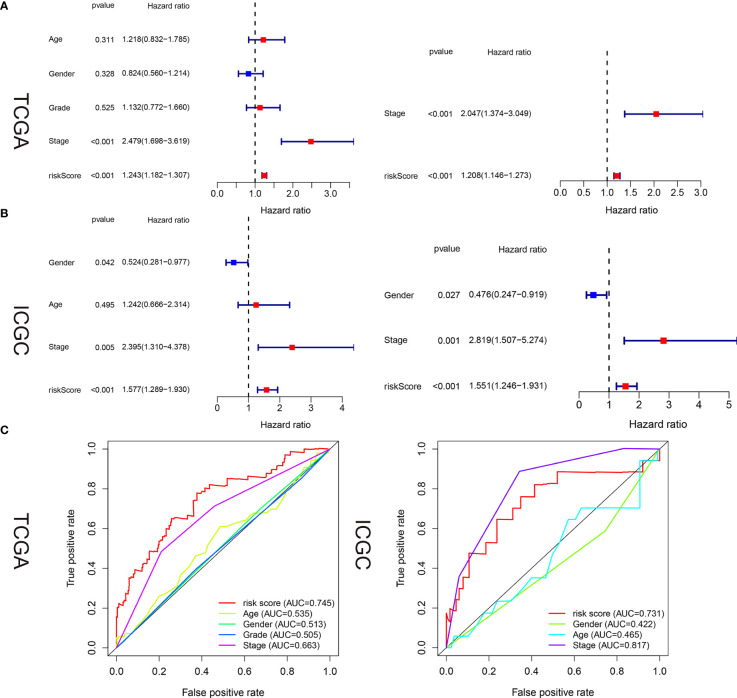
Estimated prognostic accuracy of the starvation-related mRNAs prognostic signature and other clinicopathological variables in HCC patients in the training and validation cohorts. **(A, B)** In the training and validation cohorts, univariate and multivariate analyses were performed for risk scores and each clinical feature. **(C)** Time-dependent ROC curve of risk scores and clinical features were predicted in the training and validation sets at 5 years.

Univariate and multivariate analyses were used to determine whether our hunger-related gene model could be an independent prognostic factor. In TCGA, risk scores and sex, age, grade, and stage of starvation-related gene-building models were used for univariate and multivariate analyses. In the univariate analysis, stage (HR = 2.479, 95% CI 1.698–3.619, P < 0.001) and risk score (HR = 1.243, 95% CI 1.182–1.307, P < 0.001) were associated with OS. In the multivariate analysis, stage (HR = 2.047, 95% CI 1.374–3.049, P < 0.001) and risk score (HR = 1.208, 95% CI 1.146–1.273, P < 0.001) were associated with OS (**Figure 5A**). In the ICGC, the risk scores of the starvation-related gene-building prognostic models and clinical characteristics such as age, sex, and stage were also analyzed by univariate and multivariate analyses. The results of the univariate analysis showed that gender, stage, and risk scores were associated with OS. The results of the multivariate analysis showed that gender, stage, and risk scores were also associated with OS (**Figure 5B**).

In the training and validation cohorts, it is evident that the starvation-related gene prognostic model we constructed can act as an independent prognostic factor in patients with liver cancer. areas under the curve (AUC) values in the training and validation sets were 0.745 and 0.731, respectively, indicating high accuracy of the risk score as an independent prognostic factor (**Figure 5C**).

### Identification of Differential Starvation-Related Genes Associated With Prognosis in Patients With HCC

**Figure 6:**
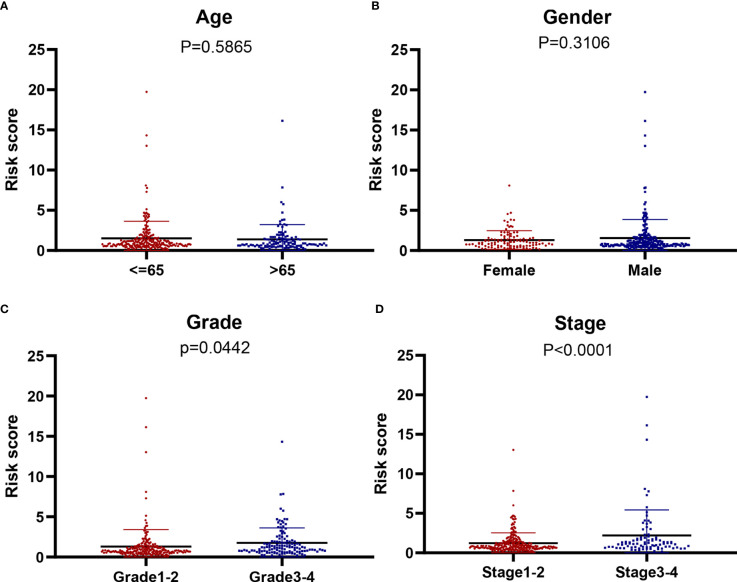
The correlation of our signature with the clinicopathological characters of HCC. **(A)** Age (≥ 65 *vs.* < 6 5 years; P = 0,5865), **(B)** gender (male *vs.* female; P = 0.3106), **(C)** tumor grade (grade 1-2 *vs.* 3-4; P = 0.0442) **(D)** tumor stage (stage 3-4 *vs.* 1-2; P < 0.001).

We further analyzed the relationship between the model of genes related to starvation and clinical characteristics. It is evident that the model is not related to age and gender (**Figures 6A, B**) but is closely related to the liver cancer grade and liver cancer stage (**Figures 6C, D**). The higher the stage and grade of the patients with increased risk value, the higher the model of hunger-related gene construction and HCC progression were closely related.

### Stratified Analysis

**Figure 7:**
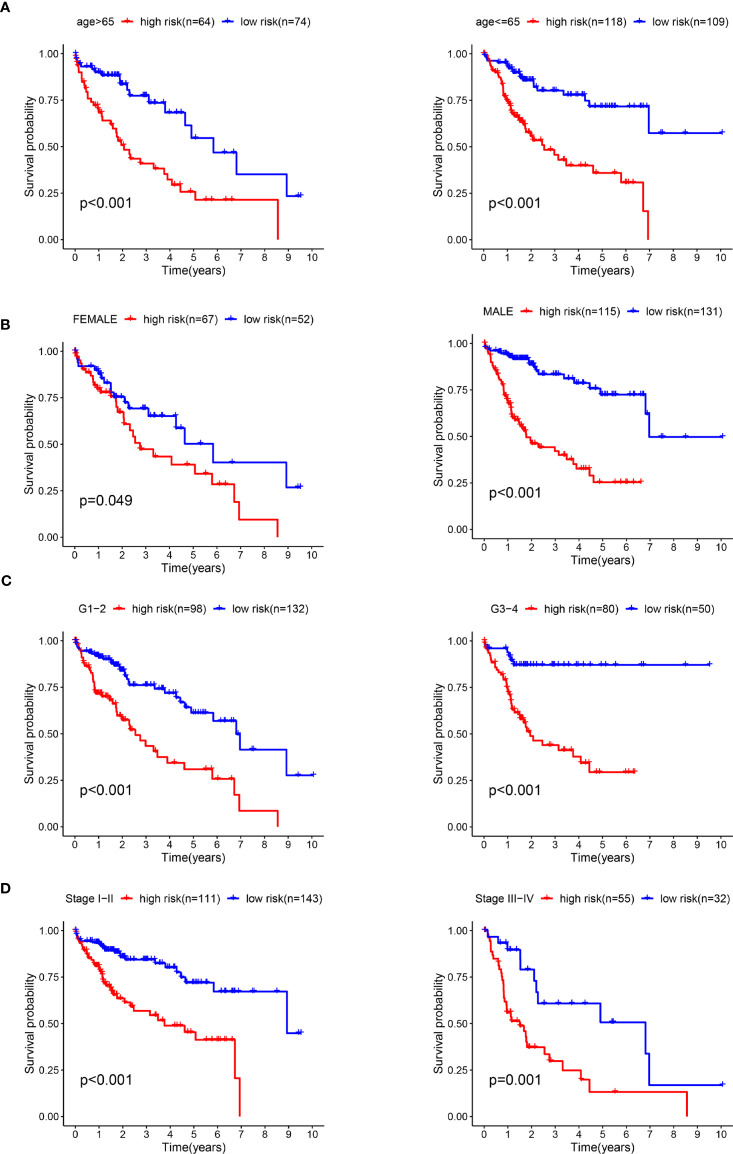
The survival rates of high- and low-risk HCC patients stratified by different clinicopathological characteristics. Kaplan-Meier survival curve analysis shows overall survival (OS) rates of high- and low-risk HCC patients from the TCGA database stratified by age (≤ 65 *vs.* > 65) **(A)**, gender (male *vs.* female) **(B)**, tumor grades (high grade *vs.* low grade) **(C)**, stages (stages I and II *vs.* stages III and IV) **(D)**.

We conducted a stratified analysis of age, sex, staging, and grading to verify the accuracy of our model. We divided the patients into low- and high-risk groups based on the median risk score. The results showed that our model had excellent predictive significance at ages > 65 years and < 65 years for both males and females, grade 1–2 and grade 3–4, stage 1–2 and levels 3-4 (**Figures 7A**–**D**).

### Establishment of a Nomogram Based on Starvation-Related Genes

**Figure 8:**
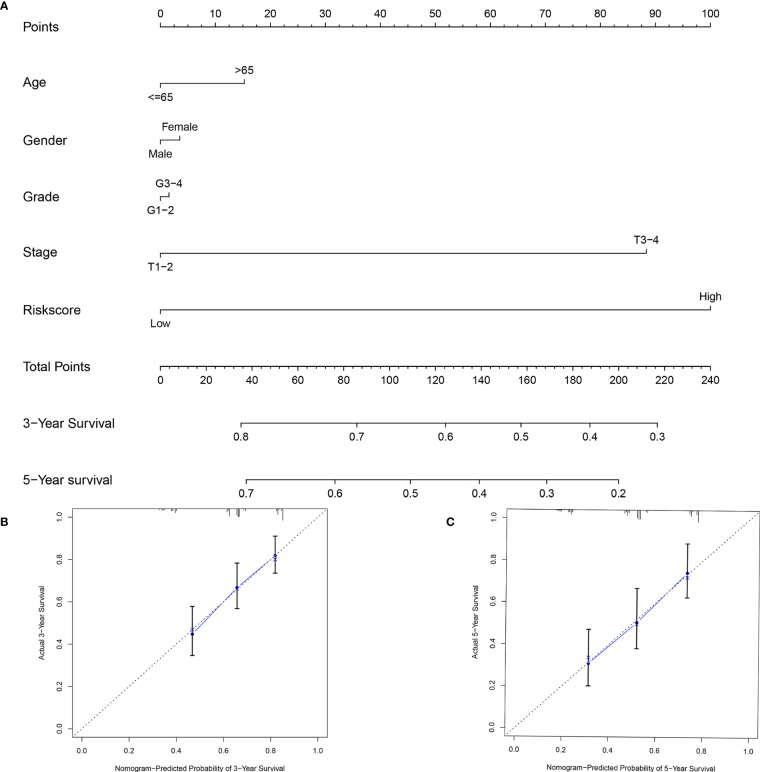
An established nomogram for predicting OS. **(A)** Construction and validation of the prognostic nomogram with starvation-related mRNA prognostic signature risk score as one of the parameters in TCGA. Calibration curve of the nomogram for the prediction of 3- **(B)** and 5-year OS **(C)**.

To provide clinicians with a practical clinical tool for predicting 3-year and 5-year OS incidence in liver cancer patients, we constructed a nomogram based on clinicopathological characteristics (age, sex, grade, stage) and risk score based on the 9-mRNA signature (**Figure 8A**). The 3-year and 5-year overall survival (OS) calibration curve is a better predictor than the ideal model (**Figures 8B, C**).

### Analysis of Biological Processes Associated With Starvation-Related Genes

**Figure 9:**
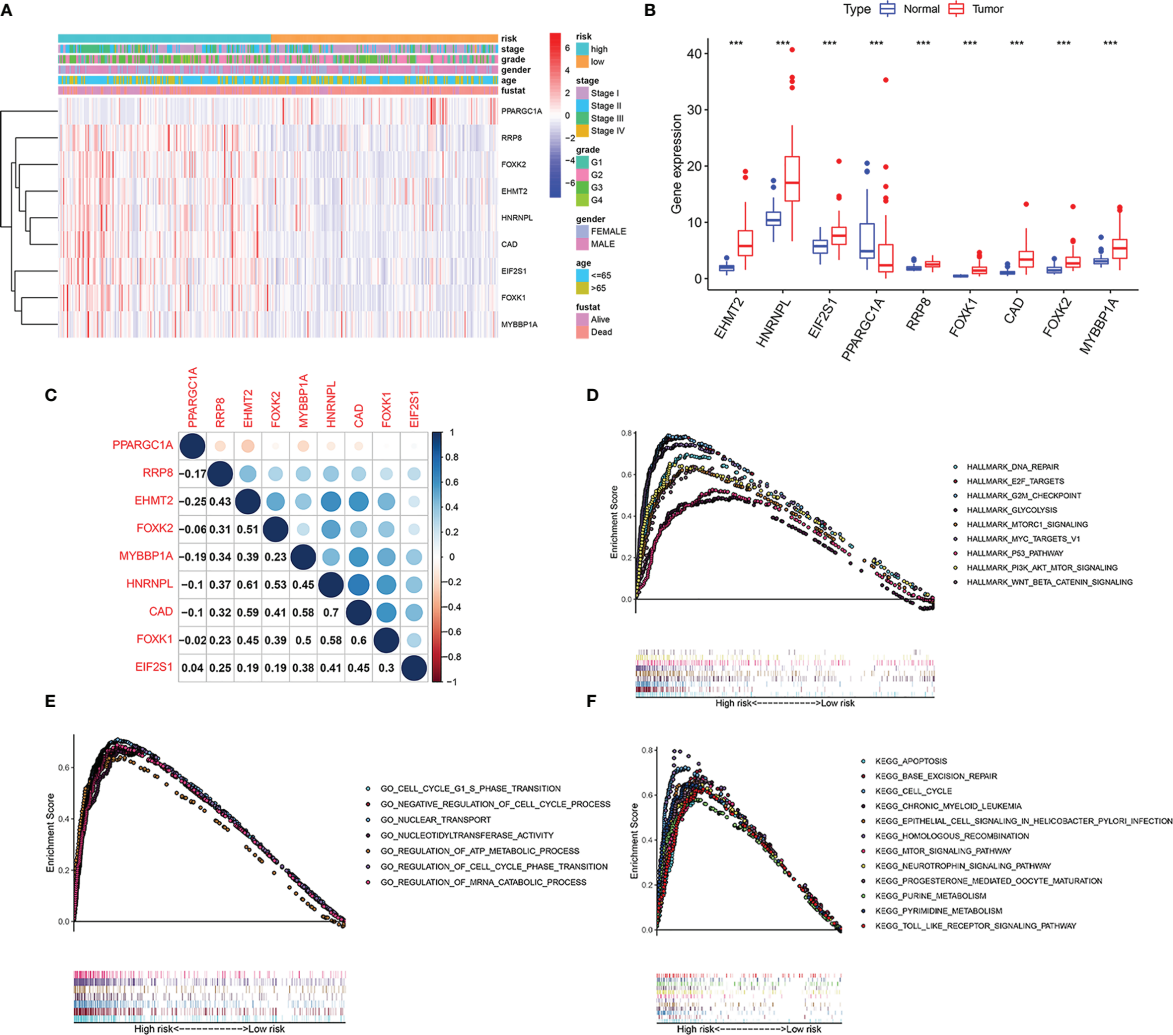
Identification of the 9 starvation-related genes. **(A)** Risk factor score, clinical features, and expression of 9 mRNAs in each patient. **(B)** Expression of 9 mRNAs in hepatocellular carcinoma and its adjacent tissues. ***P < 0.001 vs adjacent tissues. **(C)** Correlation between 9 mRNAs. **(D)** Hallmark, **(E)** GO, and **(F)** KEGG associated with signature-based risk score were performed by GSEA with nominal P-value < 0.05.

The expression level of EHMT2, HNRNPL, EIF2S1, RRP8, FOXK1, CAD FOXK2, and MYBBP1A increased with an increase in the risk coefficient of the patient, while the expression level of PPARGC1A decreases with an increase in the risk coefficient (**Figure 9A**). The expression level of EHMT2, HNRNPL, EIF2S1, RRP8, FOXK1, CAD FOXK2, and MYBBP1A was significantly higher in cancer, while the expression of PPARGC1A was lower in liver cancer (**Figure 9B**). **Figure 9C** shows the correlation of genes in the nine models. CAD and HNRNPL had the strongest positive correlation, while PPARGC1A and EHMT2 had the strongest negative correlation. GSEA was conducted to identify starvation-related biological processes and carcinogenic signaling pathways. The results revealed that “Hallmark analysis” gene sets involving cell cycle signals, PI3K/AKT/mTOR pathway, glycolysis, and p53 pathways related to cancer biological processes were enriched in the high-risk group. In addition, several typical pathways from the GO and KEGG genomes, including cell cycle pathways, mTOR signaling pathways, and apoptotic responses, were highly enriched in high-risk phenotypes (**Figures 9D–F**).

### Knockdown of EIF2S1 Inhibits Cell Invasion and Migration in HCC

**Figure 10:**
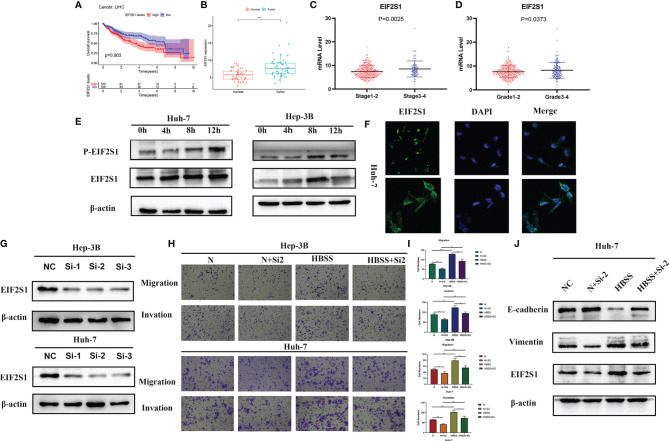
Effect of EIF2S1 on cell migration and invasion in HCC cells. **(A)** Kaplan-Meier curves show survival outcomes in patients with high and low EIF2S1 expression. **(B)** EIF2S1 expression in hepatocellular carcinoma and its adjacent tissues. **(C, D)** EIF2S1 expression in early and advanced HCC. **(E, F)** EIF2S1 expression in 3B and Huh-7 cells was induced by starvation. **(G)** Western blotting was used to detect the expression of EIF2S1 in siRNA-transfected huh-7 and 3B cells. **(H, I)** Transwell assay was used to assess the starvation-induced migration and invasion of hepatocellular carcinoma cells after EIF2S1 transfection (100× magnification). *P < 0.05, **P < 0.01 and ***P < 0.001 *vs* NC (Huh-7) or NC (Hep-3B). **(J)** Western blots showing the levels of the EIF2S1, E-cadherin, and vimentin proteins.

As shown in **Figures 10A, B**, EIF2S1 expression level in liver cancer is increased and is closely related to the degree of malignancy and prognosis of the disease. Thehigher the expression level of EIF2S1, the worse the prognosis of patients. It can be seen from **Figures 10C, D** that EIF2S1 expression levels were higher in HCC patients with high stage or grade HCC. EIF2S1 expression and phosphorylation levels were higher when HCC cells 3B and Huh-7 were in the starvation state (**Figures 10E, F**). **Figure 10G** further demonstrated that starvation could increase EIF2S1 expression in HCC cells Huh-7. Hunger and false hunger can induce cancer metastasis (10). We knocked down the expression levels of EIF2S1 in HCC cells 3B and Huh-7 (**Figure 10H**). EIF2S1 knockdown can reduce the invasive and metastatic ability of HCC cells 3B and Huh-7, both under starvation and normal conditions (**Figure 10I**). In addition, upon EIF2S1 knockdown in Huh-7 cells, the protein expression of E-cadherin increased while that of Vimentin decreased, suggesting that EIF2S1 may affect the invasion and metastasis ability of HCC cells through EMT (**Figure 10J**).

## Discussion

An increasing amount of evidence indicates that the interaction between the tumor microenvironment and tumor cells is closely related to the occurrence and development of tumors (15). Wang et al. reported that stromal components of liver cancer contribute to the malignant progression of cancer by stimulating proliferation, migration, and invasion of cancer cells and activating angiogenesis (16). Due to dysregulation of cancer growth metabolism and inadequate nutrient supply, especially glucose deficiency, nutritional deprivation in cancer is a common condition in the tumor microenvironment (17). In bladder cancer, hunger leads to autophagy, increasing cancer cell invasive and metastatic potentials (13). In oral squamous cell carcinoma (OSCC), the expression of the glycolytic enzyme phosphofructokinase-platelets (PFKP) is significantly elevated under starvation conditions, and PFKP knockdown inhibits starvation-mediated glycolysis, autophagy, and EMT in OSCC cells, thus promoting the malignant progression of OSCC (18). Glucose starvation can promote apoptosis of the TNBC cell line MDA-MB-231 and reduce its migration potential, therefore, suggesting a role for nutritional restriction in carcinogenesis (19). Starvation induces autophagy to capture and degrade intracellular proteins and organelles in lysosomes, recycling intracellular components to fuel metabolism and survival (20). Autophagy is closely related to drug resistance, stem cell resistance, and EMT in cancer (21–23). Increasing evidence shows that hunger is closely related to the occurrence and development of cancer.

With the limitation of a single gene as a prognostic factor, an increasing number of studies have shown that mRNA-constructed models can be a good independent prognostic factor for cancer. In pancreatic ductal adenocarcinoma, the model constructed using 6-mRNAs can be an independent prognostic factor and is closely related to the grade of pancreatic ductal adenocarcinoma (24). In HCC, Xie et al. described a new model comprising seven gene compositions closely related to patient prognosis (25). Another four-gene model is a good predictor of survival in patients with lung adenocarcinoma with lymph node metastasis (26). Xie et al. identifiedfour models of metastasis-related gene composition as good prognostic factors for breast cancer patients (27). Wu et al. constructed a model of nine genes in renal cell carcinoma that could predict the prognosis of stage III clear cell renal cell carcinoma (28).

Furthermore, cancer cells reprogram their metabolism to sustain their rapid growth; Zhang et al. analyzed the mechanisms underlying dysregulated glucose metabolite-related pathways in HCC to identify diagnostic, prognostic, or therapeutic targets for HCC (29). Therefore, a starvation-related mRNA signature may be a new marker for liver cancer malignancy and a potential indicator of prognosis in liver cancer patients.

Here, we first constructed a model with nine hunger-related genes and verified the model accuracy through an external cohort. Our study shows that our model is associated with the malignant progression of HCC and can act as an independent prognostic factor. We constructed a nomogram composed of models and clinical features to predict the prognosis of patients and verified the accuracy of the nomogram prediction. We further analyzed the core gene EIF2S2 for protein interaction in the nine modeled genes. *In vitro* experiments showed that the expression and phosphorylation of EIF2S1 were significantly increased following starvation induction. After EIF2S1 expression was inhibited, the invasion, and metastatic ability of HCC cells were lower under starvation.

We divided patients into high- and low-risk groups according to the median risk value. Through GSEA, we found that the MTORC1 and cell cycle-related pathways were significantly enriched in high-risk patients. The MORTC1 signal pathway is closely related to cell metabolism, growth, and autophagy (30). MTORC1 can promote the transport, processing, and synthesis of SREBPs (a family of important transcription factors for lipid synthesis), thus playing an important role in promoting fat formation (31). Rapamycin complex 1 (mTORC1) maintains cell homeostasis by linking environmental cues, including the use of nutrients in glioblastoma; hunger induces autophagy that forces tumor cells in the G1 phase, leaving them in a resting state. These factors enhance glioblastoma cell survival and chemical resistance (32). We speculate that a similar mechanism might be at play in HCC; however, this must be verified experimentally.

Growth factors and metabolic process-related stress might further amplify the perceived fluctuations in extracellular and intracellular nutrients, thereby regulating cell growth, metabolism, and survival (33). Of note, EIF2S1 plays an important role in protein translation initiation (10), and its expression is significantly increased after chemotherapy in breast cancer patients. It can promote the survival of breast cancer cells during chemotherapy (34). EIF2S1 interacts with TOR signaling modulator-like (TIPRL) proteins to induce autophagy and enhance lung cancer malignancy (35). In our study, we found that starvation induction can promote the expression of EIF2S1 and P-EIF2s1 in HCC. EIF2S1 can affect the invasion and metastasis ability of liver cancer.

In conclusion, a nine starvation-related mRNA signature correlated with HCC progression and prognosis and could be used as independent prognostic molecular biomarkers for predicting HCC survival.

## Data Availability Statement

The original contributions presented in the study are included in the article/**Supplementary Material**. Further inquiries can be directed to the corresponding author.

## Author Contributions

DL developed the study concept and design, performed data acquisition and analysis, and drafted the manuscript. YC performed the bioinformatics and statistical data analyses. YZ and GH constructed the figures and tables. FL was responsible for the integrity of the entire study and manuscript review. All authors contributed to the article and approved the submitted version.

## Funding

This study was supported by the National Natural Science Foundation of China (No. 81372481).

## Conflict of Interest

The authors declare that the research was conducted in the absence of any commercial or financial relationships that could be construed as a potential conflict of interest.

## Publisher’s Note

All claims expressed in this article are solely those of the authors and do not necessarily represent those of their affiliated organizations, or those of the publisher, the editors and the reviewers. Any product that may be evaluated in this article, or claim that may be made by its manufacturer, is not guaranteed or endorsed by the publisher.
